# When to initiate immunomodulatory therapy in sepsis

**DOI:** 10.3389/fimmu.2026.1898450

**Published:** 2026-07-29

**Authors:** Yi Wen, Dan Huang

**Affiliations:** 1 Department of Critical Care Medicine, Chengdu Medical College The First Affiliated Hospital, Chengdu, China; 2 School of Clinical Medicine, Chengdu Medical College, Chengdu, China

**Keywords:** biomarkers, immunomodulation, immunophenotype, sepsis, timing

## Abstract

Immunomodulatory therapy in sepsis has long failed to deliver consistent clinical benefit, in part because the heterogeneity of host responses and the dynamic nature of disease trajectories have been underestimated. Rather than equating the timing of initiation with a fixed interval after symptom onset, a more clinically meaningful definition is a chronology-informed clinical decision point at which standard sepsis care has been implemented, serial monitoring supports a dominant or clinically important discordant immunophenotype, and the organ dysfunction trajectory suggests that continued observation without reassessment or escalation may be unsafe. Using this operational definition as its framework, this review synthesizes the dynamic course of immune dysregulation in sepsis, the clinical utility and limitations of key biomarkers and functional assays, and the emerging evidence from enrichment-design trials that reassess the therapeutic windows of different strategies. Current evidence suggests that intravenous corticosteroids in adult septic shock remain the immunomodulatory approach closest to routine clinical practice, whereas anakinra, granulocyte-macrophage colony-stimulating factor (GM-CSF), interferon-gamma (IFN-gamma), interleukin-7 (IL-7), and programmed cell death protein 1/programmed death-ligand 1 (PD-1/PD-L1) blockade are more appropriately considered in biomarker-enriched settings with rigorous serial reassessment, ideally within research protocols or experienced centers. The central challenge for the future is not to identify a universal immune drug for all patients with sepsis, but to establish reproducible, bedside-applicable immune phenotyping and reassessment pathways that can determine who may benefit, from which intervention, and at what point in the disease course.

## 1 Introduction

Sepsis is not simply a synonym for an inflammatory storm; it is life-threatening organ dysfunction triggered by infection and fundamentally defined by a dysregulated host response (1). The Sepsis-3 framework removed systemic inflammatory response syndrome (SIRS) from the diagnostic criteria for sepsis, and any contemporary review must therefore distinguish rigorously among infection, SIRS, sepsis, and septic shock. SIRS may arise from infectious or non-infectious causes, whereas sepsis requires both an infectious context and organ dysfunction (1). Much of the current controversy surrounding immunomodulatory therapy does not stem from the absence of potentially effective agents, but from the fact that most interventions have not been applied in the right patients, at the right stage, or against the right targets (2–4).

Recent advances in sepsis immunobiology further support the view that host responses are best understood as a dynamic disruption of immune homeostasis. A high inflammatory burden may predominate early, followed by immunosuppression and immunoparalysis, but these states frequently coexist rather than occurring in a simple linear sequence (3–5). This immunodynamic disorder helps explain why many prior anti-inflammatory, immune-enhancing, and immune-checkpoint strategies yielded inconsistent results in unselected populations and may even have worsened outcomes when delivered at the wrong biological moment (5, 8).

Several recent reviews have summarized immunotherapy in sepsis or discussed precision medicine from a broad mechanistic perspective (3, 4, 9). The present review differs by focusing on a narrower and clinically actionable question: when can a specific immunomodulatory strategy reasonably be considered. Its central contribution is an operational framework that integrates chronological disease evolution, response to standard care, serial immune assessment, organ dysfunction trajectory, and evidence strength. This approach is intended to move the discussion from a drug-centered catalogue toward a bedside decision framework for selected patients.

## 2 Chronology-informed immune dynamics and initiation timing in sepsis

The timing of immunomodulatory therapy should not be reduced to a fixed number of hours after sepsis onset. Sepsis is nevertheless a time-dependent syndrome, and chronological disease evolution remains an essential clinical framework because host immune responses often show recognizable temporal patterns despite substantial interindividual heterogeneity. Initiation timing should therefore be defined as a chronology-informed clinical decision point that integrates the stage of disease evolution, the response to standard care, serial evidence supporting a dominant immunophenotype, and the trajectory of organ dysfunction. In this context, timing is determined by chronology-informed clinical context, phenotypic evidence, and dynamic change rather than by a single biomarker value or a rigid time stamp (2–4). The 2026 Surviving Sepsis Campaign (SSC) guidelines reflect this cautious position by supporting intravenous corticosteroids in adult septic shock while not recommending most other immune-related interventions for routine use (2).

**Figure 1:**
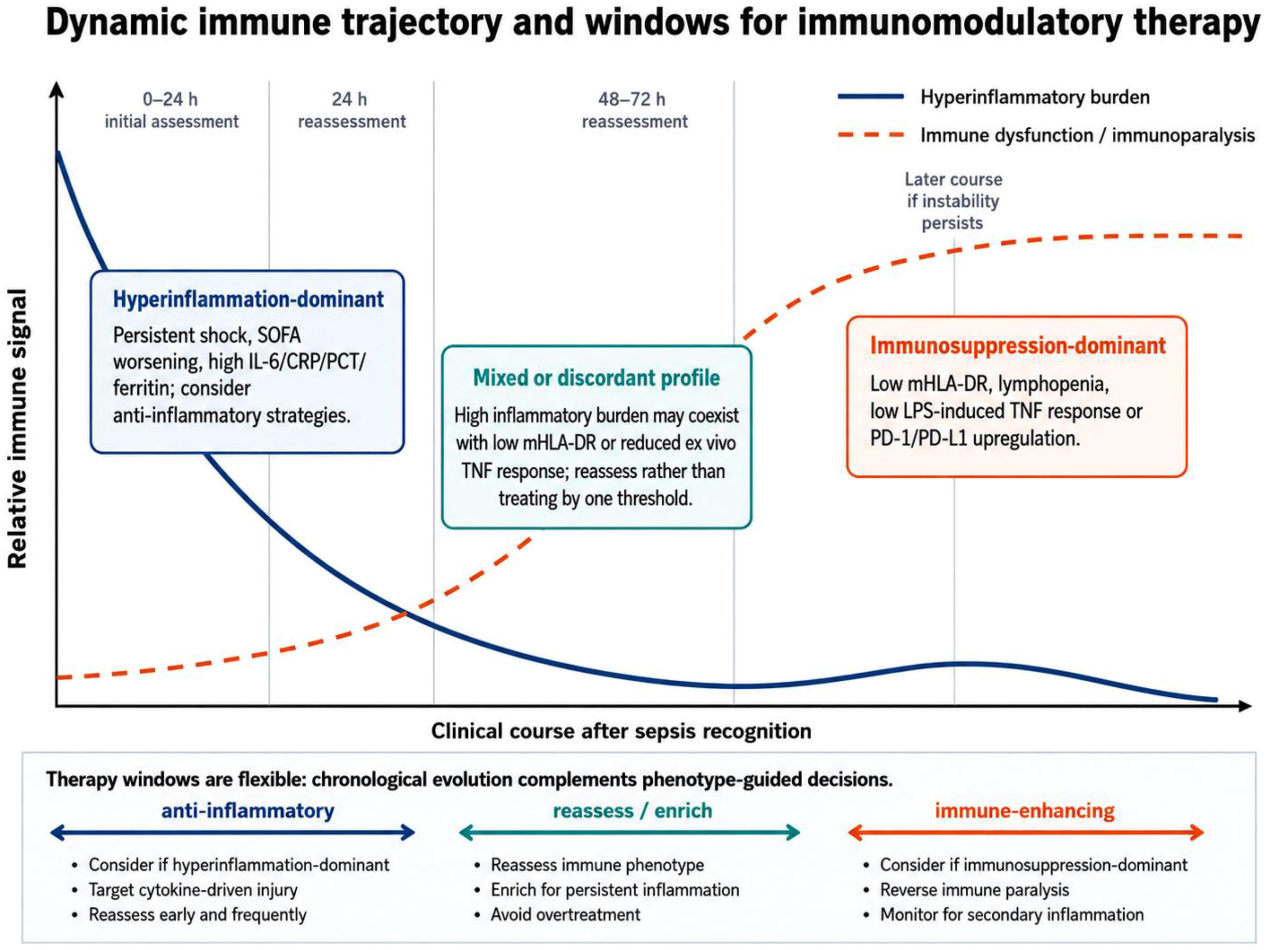
Dynamic trajectory of immune dysregulation in sepsis and potential windows for immunomodulatory therapy. The figure emphasizes that chronological evolution complements immune phenotyping. Baseline assessment, 24-h reassessment, and 48-72-h reassessment are flexible decision checkpoints rather than rigid treatment thresholds.

A high inflammatory burden is commonly observed during the first several hours to days of illness, whereas persistent immune dysfunction often becomes more evident later in the clinical course. These temporal patterns should be interpreted as clinical tendencies rather than fixed biological phases. Some patients already exhibit concurrent hyperinflammation and immunosuppression at intensive care unit (ICU) admission, while others transition rapidly toward immune dysfunction after initial hemodynamic stabilization (3–5). The simplified model in which 24–72 h represents an inflammatory phase and days 3–7 represent an immunosuppressive phase is therefore insufficiently rigorous for guiding immunomodulatory therapy. A more clinically meaningful approach is to infer the dominant immune state from repeated assessments. Persistent shock, worsening Sequential Organ Failure Assessment (SOFA) score, and a rising inflammatory burden support a hyperinflammation-dominant state, whereas sustained lymphopenia, persistently reduced monocyte human leukocyte antigen-DR (mHLA-DR) expression, secondary infection, and delayed clinical recovery are more consistent with immunosuppression or immunoparalysis (**Figure 1**).

Mechanistic studies in related cytokine-storm models further illustrate the stage-dependent role of macrophages. In a murine model of chimeric antigen receptor T-cell-induced cytokine release syndrome (CRS), recipient macrophages were major sources of interleukin-1 and interleukin-6, and interleukin-1 blockade attenuated systemic toxicity (6). In an infection-induced murine cytokine storm syndrome (CSS) model, macrophage depletion at disease onset prolonged survival, whereas delayed depletion after disease establishment worsened severity (7). These models are not equivalent to human sepsis and do not provide direct clinical evidence for macrophage-targeted treatment. They nevertheless demonstrate that macrophage composition and the consequences of macrophage-directed intervention may change over time, reinforcing the need to interpret immune phenotype within the chronological stage of illness.

## 3 Literature selection and evidence prioritization

This narrative review was developed through a targeted literature search of PubMed, MEDLINE, Web of Science, and major journal websites. Searches focused on studies published from 2000 to 2026, with additional older landmark studies included when they established key concepts or trial evidence. Search terms included sepsis, septic shock, immunomodulation, immunotherapy, corticosteroids, anakinra, IL-1 blockade, GM-CSF, IFN-gamma, IL-7, PD-1, PD-L1, mHLA-DR, ferritin, lymphopenia, endotoxin tolerance, LPS-stimulated TNF-alpha, immune phenotyping, biomarker enrichment, and precision medicine.

Evidence was prioritized according to clinical relevance and methodological strength. International guidelines, randomized trials, enrichment-design trials, systematic reviews, and high-quality translational studies in human sepsis were given the greatest weight. Mechanistic and animal studies were used only when they clarified biological plausibility or helped interpret functional immune assays. Because this is a narrative review rather than a systematic review, no formal risk-of-bias scoring was performed. The selection strategy was designed to support a clinically oriented synthesis of timing, immunophenotype, and evidence strength rather than an exhaustive catalogue of all immunomodulatory interventions.

## 4 Immune-phenotype assessment: biomarkers, functional assays, and clinical feasibility

The tools currently used to assess immune phenotypes in sepsis can be grouped into biomarkers reflecting inflammatory burden, biomarkers associated with immunosuppression, and functional or endotype-oriented assays. C-reactive protein (CRP), procalcitonin (PCT), interleukin-6 (IL-6), and ferritin are widely available and can help identify high-inflammatory states, but they are not specific to sepsis, and their absolute values are strongly influenced by infection site, pathogen type, comorbidities, and sampling time (3, 8, 9). Emerging markers, including monocyte distribution width, presepsin, soluble triggering receptor expressed on myeloid cells-1 (sTREM-1), and related myeloid or endothelial markers, may improve risk stratification in selected settings, although they are not yet sufficiently standardized for routine phenotype-guided initiation of immunomodulatory therapy. In recent precision-immunotherapy trials, ferritin has extended beyond general inflammatory monitoring because it has been used to identify hyperinflammatory subgroups with macrophage activation-like syndrome (MALS) features. PROVIDE and ImmunoSep used ferritin higher than 4420 ng/mL as a research enrichment criterion (10, 11). This value should be interpreted as a trial-enrichment threshold rather than a universally validated bedside cutoff.

mHLA-DR is among the most representative biomarkers of sepsis-associated immunosuppression. Persistently low expression suggests impaired monocyte antigen presentation and has been associated with secondary infection and adverse outcomes (12). Its implementation remains constrained by differences in analytical platforms, unit conversion, and the external validity of published thresholds. Early studies and subsequent enrichment trials have not used identical cutoffs. The Meisel randomized trial used less than 8000 mAb/cell as an enrollment criterion, whereas PROVIDE and ImmunoSep used less than 5000 HLA-DR receptors per monocyte to identify immunoparalysis (10, 11, 13). The work by Demaret et al. also showed that strict inter-laboratory standardization is required when flow-cytometric platforms are used (14). mHLA-DR is therefore best described as a valuable but standardization-dependent enrichment biomarker rather than as a single threshold that can be generalized across settings.

Peripheral lymphocyte count, programmed cell death protein 1/programmed death-ligand 1 (PD-1/PD-L1) pathway markers, and functional immune assays provide additional information about adaptive and innate immune status. Persistent lymphopenia is accessible but lacks specificity. PD-1 and PD-L1 are mechanistically closer to T-cell exhaustion and checkpoint-mediated suppression, yet soluble measurements are not interchangeable with cell-surface expression and remain largely research tools (15, 16). Low mHLA-DR, lymphopenia, checkpoint activation, and reduced ex vivo cytokine production reflect overlapping but biologically distinct dimensions of immune dysfunction. They should not be collapsed into a single label of immunosuppression without considering whether the dominant abnormality is impaired antigen presentation, innate immune hyporesponsiveness, adaptive exhaustion, or broader immune paralysis.

Functional assays are particularly relevant to the question of treatment timing because they evaluate immune-cell responsiveness rather than merely measuring circulating marker abundance. lipopolysaccharide (LPS)-stimulated tumor necrosis factor-alpha (TNF-alpha) release and related whole-blood or monocyte stimulation assays directly assess Toll-like receptor (TLR)-mediated innate immune capacity. Transcriptomic and mechanistic studies in human sepsis have shown that monocytes can undergo functional reprogramming with endotoxin-tolerance-like features, mediated by pathways involving hypoxia-inducible factor-1alpha (HIF-1alpha), NFkB2/p100, p21-dependent nuclear factor-kappa B (NF-kappaB) rewiring, and PD-L1 upregulation during endotoxin tolerance (17–21). Mathematical modeling of monocyte and whole-body responses to endotoxin further supports the concept that inflammatory and tolerant states are dynamic, stimulus-dependent, and time-sensitive rather than static categories (22).

Clinical evidence for ex vivo stimulation assays is growing but remains heterogeneous. A systematic review of LPS-induced TNF-alpha production found that lower TNF-alpha responses tended to correlate with higher mortality, although methodological differences in stimulant dose, incubation time, sample processing, and outcome definitions prevented firm conclusions (23). More recent clinical work suggested that reduced TNF-alpha production after LPS challenge may help identify sepsis-induced immunosuppression and predict positive blood cultures, reinforcing the potential value of functional immune profiling (24). These assays are biologically attractive because they link immune phenotype to cellular function, but they remain limited by pre-analytical variability, turnaround time, cost, and lack of platform standardization. In most real-world ICUs, the most feasible strategy is a layered approach combining clinical course, routine inflammatory markers, lymphocyte count, and, where available, mHLA-DR or functional testing, rather than postponing decisions until complex multi-omic results become available.

**Supplementary Table 1** summarizes the clinical value, limitations, and feasibility of major immune-phenotyping tools. At present, trends are generally more informative than isolated thresholds. For decisions regarding immunomodulatory therapy, baseline assessment at ICU admission, repeat testing at 24 h and 48–72 h, and additional testing when clinical status changes abruptly are usually more informative than a single sample.

## 5 Phenotype-informed initiation and management of discordant immune profiles

**Figure 2:**
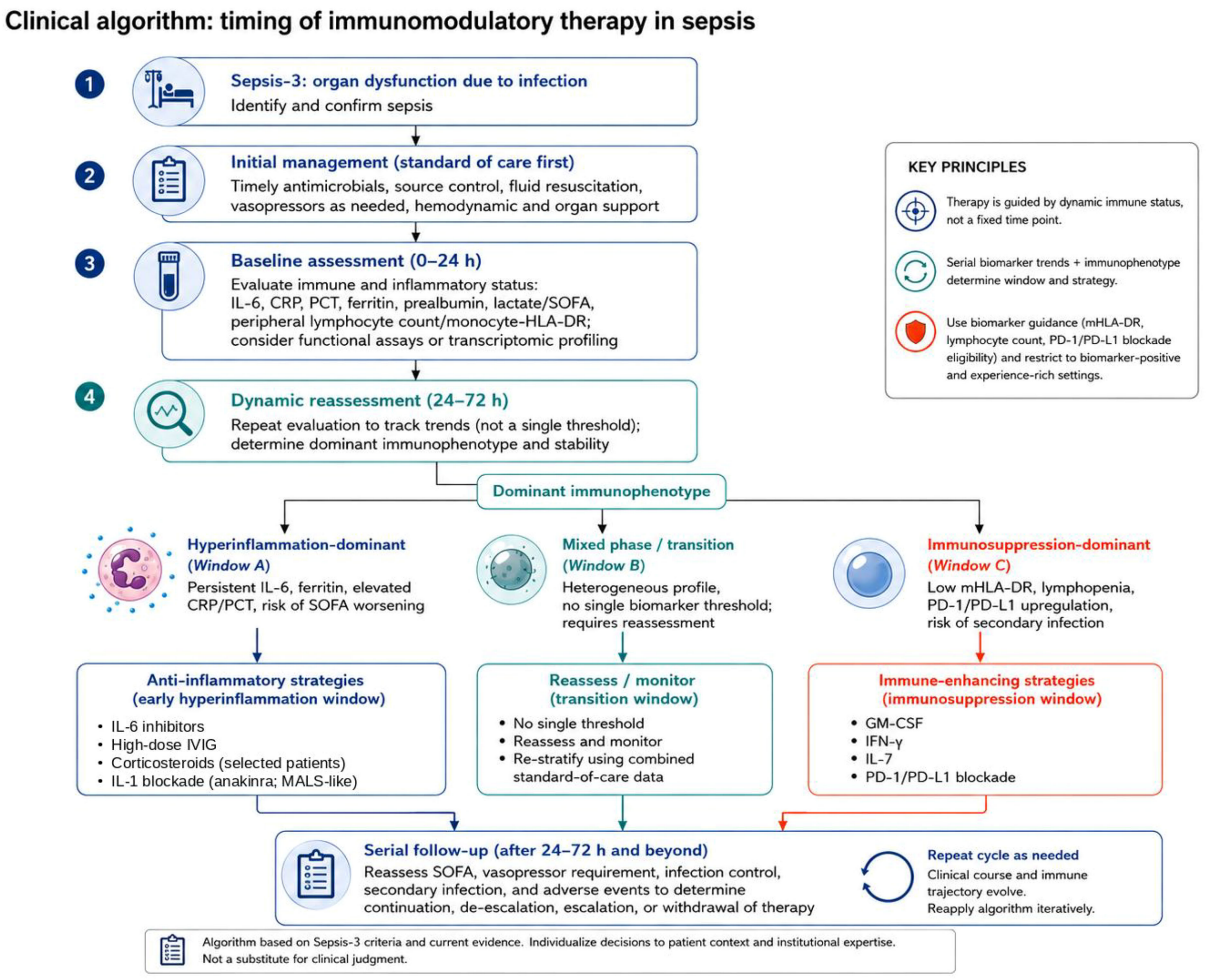
Proposed algorithm for initiating immunomodulatory therapy according to immune phenotype. The algorithm emphasizes a closed-loop strategy in which standard care is implemented first, immune phenotyping is interpreted alongside clinical chronology, and reassessment after initiation guides continuation, de-escalation, escalation, or withdrawal. Within the hyperinflammation branch, IL-1 blockade with anakinra is limited to selected MALS-like phenotypes.

Before immunomodulatory therapy is considered, standard sepsis care must have been appropriately implemented and reassessed, including timely antimicrobial therapy, source control, adequate but judicious fluid resuscitation, hemodynamic support, and organ support (2). Uncontrolled infection, inadequate antimicrobial coverage, unresolved hypovolemia, or insufficient organ support may mimic immune failure and should not be mistaken for an indication for immune-directed treatment. Immunomodulation becomes clinically meaningful only when the ongoing clinical trajectory cannot be explained by correctable deficiencies in standard care, and when serial assessment supports either a reproducible dominant immunophenotype or a clinically important discordant immune profile. **Figure 2** presents a practical algorithm for this decision process.

In hyperinflammation-dominant states, the most defensible near-practice strategy remains corticosteroid therapy in selected adults with septic shock and persistent hemodynamic instability. The ADRENAL trial did not show a clear mortality benefit but demonstrated faster shock reversal, whereas APROCCHSS reported reduced 90-day mortality (25, 26). VANISH further highlighted the interaction between hemodynamic management and corticosteroid exposure, supporting the view that corticosteroids should be considered in patients with ongoing septic shock rather than administered empirically to all patients with sepsis (27). The timing of corticosteroid initiation should therefore be anchored primarily to persistent vasopressor-dependent shock after appropriate initial resuscitation rather than to cytokine concentrations alone. Other anti-inflammatory strategies require stronger biological enrichment. Early phase III trials of recombinant human interleukin-1 receptor antagonist in broadly enrolled sepsis populations did not establish a definitive overall survival benefit, arguing against empirical use in unselected patients (28, 29). Subsequent evidence suggests that potential benefit may be concentrated in biologically enriched subgroups. The *post hoc* analysis by Shakoory et al. identified a survival signal in patients with concurrent hepatobiliary dysfunction and disseminated intravascular coagulation consistent with a MALS-like phenotype, whereas PROVIDE and ImmunoSep used marked hyperferritinemia to identify patients for anakinra treatment (10, 11, 30). Interleukin-1 blockade should therefore be positioned as a biomarker-supported investigational strategy for selected MALS-like hyperinflammation rather than as routine initial anti-inflammatory therapy.

In immunosuppression-dominant states, immune-enhancing interventions are biologically plausible but remain largely investigational. Their potential timing is most relevant in patients with delayed recovery despite adequate source control, recurrent or secondary infection, persistent organ-support requirements, sustained lymphopenia, reduced mHLA-DR expression, low LPS-induced TNF-alpha response, or checkpoint activation (12–14, 23, 24, 31). The biomarker-guided granulocyte-macrophage colony-stimulating factor (GM-CSF) trial by Meisel et al. showed recovery of monocyte immune function in patients selected on the basis of low mHLA-DR expression (13). Interleukin-7 (IL-7) studies, including IRIS-7 and subsequent intravenous IL-7 work, demonstrated increases in lymphocyte counts and selected immune markers, although the evidence remains mainly early phase (32, 33). PD-1 and PD-L1 blockade has also been explored in enriched patients with sepsis-associated immunoparalysis. Available phase I and II studies suggest that anti-PD-L1 antibodies and nivolumab did not generate an obvious cytokine-storm-like safety signal and may be associated with favorable immune readouts (15, 16). These studies were designed primarily to evaluate safety, pharmacokinetics, pharmacodynamics, and immune signals rather than definitive patient-centered outcomes. GM-CSF, interferon-gamma (IFN-gamma), IL-7, and PD-1/PD-L1 modulation should therefore be positioned as biomarker-supported or exploratory strategies, preferably within research protocols or experienced centers, rather than routine ICU interventions.

Mixed or discordant immune profiles require particular caution. Hyperinflammation and immunosuppression may coexist, and elevated IL-6 or ferritin can occur together with low mHLA-DR expression, lymphopenia, checkpoint activation, or reduced LPS-induced TNF-alpha release. These markers reflect overlapping but biologically distinct processes, including impaired antigen presentation, innate immune hyporesponsiveness, adaptive immune exhaustion, and broader immune paralysis. A patient with simultaneous inflammatory injury and functional immunosuppression should not be forced into a binary treatment category. In this setting, the practical response should be to reassess source control, repeat immune testing, interpret biomarker trends in relation to organ dysfunction, and seek expert input when available. Empirical escalation to a single mechanism-based immunomodulatory therapy may be harmful if it amplifies the wrong component of the immune response.

Regardless of the selected strategy, reassessment within 24–72 h after initiation is essential. This reassessment should include SOFA trajectory, vasopressor requirement, adequacy of source control, secondary or fungal infection, adverse events, and directional changes in key immune biomarkers. If the patient improves and immune or inflammatory markers move toward a more physiological range, treatment may be continued with subsequent tapering or withdrawal when appropriate. If the intervention is ineffective, adverse effects emerge, or the immune phenotype shifts in an opposing direction, treatment should be stopped or revised promptly. This initiation, reassessment, and correction loop is more consistent with the biology of precision immunomodulation than a strategy focused only on the initial starting point (**Figure 2**; **Supplementary Table 2**).

## 6 Immunomodulatory strategies and key clinical evidence

From the perspective of evidence hierarchy, immunomodulatory strategies in sepsis can be divided into three broad tiers. The first tier includes approaches closest to routine clinical practice, represented by intravenous corticosteroids in selected adults with septic shock. The 2026 SSC guidelines provide a conditional recommendation in this setting. The second tier comprises strategies with a strong biological rationale but a continuing need for phenotype enrichment to maximize benefit, including anakinra, GM-CSF, IFN-gamma, and IL-7 (10, 11, 13, 30, 32, 33). The third tier includes exploratory interventions, such as PD-1/PD-L1 blockade, which remain at the stage of early safety and pharmacodynamic signal evaluation (15, 16). **Supplementary Table 2** summarizes the key clinical evidence and evidence positioning for these strategies.

The most important recent progress has come from enrichment-design trials. PROVIDE was the first study to systematically combine ferritin and mHLA-DR for immune classification, suggesting that phenotype-driven treatment selection is more informative than applying the same strategy to all patients with sepsis (10). ImmunoSep further showed that assigning anakinra or recombinant IFN-gamma according to a macrophage activation-like hyperinflammatory phenotype or an immunoparalysis phenotype can improve organ dysfunction-related outcomes at day 9 (11). ImmunoSep should nevertheless be interpreted carefully because its positive findings mainly concern organ-function improvement rather than an established mortality benefit or immediate incorporation into standard care. This is why the present review uses the wording may be considered rather than should be routinely used.

The discussion of intravenous immunoglobulin (IVIG) requires particular restraint. An earlier Cochrane review concluded that apparent benefit became unstable when analyses were restricted to trials at low risk of bias (34), whereas a more recent meta-analysis suggested a possible mortality signal in selected formulations, particularly IgM-enriched preparations, and selected populations (35). The 2026 SSC guidelines still recommend against routine IVIG use in adult sepsis or septic shock. IVIG should therefore be framed as a strategy with conflicting evidence and possible subgroup relevance rather than one that supports routine initiation.

Large negative immunomodulation trials should also be incorporated into the overall interpretation. The phase III TESTS trial failed to show that thymosin alpha 1 reduced 28-day mortality (36). Such results remind us that biological plausibility is insufficient when phenotype enrichment, target selection, and initiation criteria are not aligned. Negative or neutral trials do not necessarily invalidate immunomodulation as a concept; they define the evidence that remains missing before routine clinical adoption.

## 7 Translational barriers, special populations, and future directions

Although the conceptual path toward precision immunotherapy in sepsis is becoming clearer, clinical translation still faces major bottlenecks. Biomarker standardization remains incomplete, with mHLA-DR providing the clearest example of variability in units, thresholds, analytical platforms, and multicenter reproducibility (12, 14). Dynamic monitoring is also costly because timing decisions depend on trends rather than isolated values, and few ICUs can routinely perform standardized flow cytometry or functional immune testing at baseline, 24 h, 48 h, and 72 h. Resource-limited ICUs face additional barriers, including limited laboratory infrastructure, delayed sample transport, lack of specialized immunology support, and difficulty repeating assays when clinical status changes. These settings require a pragmatic layered strategy that begins with accessible clinical and laboratory variables and adds specialized assays only when they are available and actionable.

There is also a substantial gap between complex multi-omic platforms and bedside applicability. Transcriptomic, proteomic, and single-cell approaches can define endotypes with high precision, yet they rarely return results within the narrow window required for acute decision-making (3, 4, 31). Artificial intelligence-assisted immune phenotyping, machine learning-based prediction models, and rapid point-of-care immune monitoring technologies may eventually bridge this gap. Their near-term role is more likely to be the conversion of high-dimensional discoveries into parsimonious, reproducible, and rapidly measurable panels than the direct deployment of complex multi-omic algorithms at the bedside. Future models will require external validation, calibration across populations, integration with sepsis workflow, transparent interpretability, and prospective testing before they can guide immunomodulatory therapy.

Special populations deserve explicit attention. Pediatric sepsis should not be treated as a simple extension of adult sepsis. The Phoenix criteria established a new framework for pediatric sepsis and septic shock (37, 38), but immunomodulation studies in children remain sparse, and available work suggests that hydrocortisone may be associated with a longer duration of multiple organ dysfunction syndrome (MODS) in pediatric immunoparalysis (39). Patients with hematological malignancy, transplant recipients, and chronically immunosuppressed individuals are equally challenging because baseline immune dysfunction, antimicrobial prophylaxis, immunosuppressive drugs, graft-related considerations, and atypical pathogen spectra may alter both immune biomarker interpretation and treatment risk. In these populations, immune phenotyping may be particularly relevant, but evidence is insufficient to support direct transfer of adult sepsis algorithms.

A pragmatic implementation pathway should proceed in layers. The first layer should use highly accessible clinical and laboratory variables for initial screening; the second, where available, should add mHLA-DR, peripheral immune phenotyping, or functional testing; and the third should treat transcriptomics, single-cell technologies, and machine-learning models as research-enhancement tools rather than mandatory gatekeepers for current care. **Supplementary Table 3** summarizes the main translational barriers and feasible countermeasures.

## 8 Limitations

This narrative review has inherent limitations. Although the literature selection was structured and clinically focused, it was not a formal systematic review and did not include meta-analysis or standardized risk-of-bias scoring. The evidence base remains heterogeneous with respect to sepsis definitions, timing of enrollment, infection source, organ dysfunction severity, immune biomarker platforms, enrichment thresholds, and clinical endpoints. Many studies evaluate immune readouts, shock reversal, or organ dysfunction rather than mortality or long-term recovery. Functional immune assays and multi-omic endotyping provide mechanistic depth, but their clinical feasibility and standardization remain limited. The framework proposed here should therefore be interpreted as a synthesis to support clinical reasoning and trial design, not as a validated bedside protocol for routine immunomodulatory therapy.

## 9 Conclusion

The central challenge in sepsis immunomodulation is not the discovery of ever more broad-spectrum immune drugs, but the accurate identification of when to start a specific intervention in a specific patient. Timing is not a fixed clock time; it is a chronology-informed decision point jointly defined, after standard care has been delivered, by the dominant or discordant immunophenotype, the trajectory of organ dysfunction, and serial immune measurements.

Based on current evidence, intravenous corticosteroids in selected adults with septic shock remain the immunomodulatory strategy closest to routine clinical practice. Anakinra, GM-CSF, IFN-gamma, IL-7, and PD-1/PD-L1 blockade are better reserved for biomarker-enriched settings with rigorous serial reassessment, ideally within research protocols or experienced centers. Evidence in children, immunocompromised hosts, transplant recipients, and patients with hematological malignancy remains insufficient, and adult pathways should not be transposed uncritically. Future studies should prioritize reproducible immune-phenotyping standards, dynamic reassessment frameworks, and simplified bedside-applicable immune testing. Only when the questions of who, when, and on what basis are answered together can immunomodulatory therapy in sepsis move from proof of concept toward precise clinical practice.

## Glossary

CRP: C-reactive protein
CRS: cytokine release syndrome
CSS: cytokine storm syndrome
GM-CSF: granulocyte-macrophage colony-stimulating factor
HIF-1alpha: hypoxia-inducible factor-1alpha
ICU: intensive care unit
IFN-gamma: interferon-gamma
IL: interleukin
IVIG: intravenous immunoglobulin
LPS: lipopolysaccharide
MALS: macrophage activation-like syndrome
mHLA-DR: monocyte human leukocyte antigen-DR
MODS: multiple organ dysfunction syndrome
NF-kappaB: nuclear factor-kappa B
PCT: procalcitonin
PD-1: programmed cell death protein 1
PD-L1: programmed death-ligand 1
SIRS: systemic inflammatory response syndrome
SOFA: Sequential Organ Failure Assessment
SSC: Surviving Sepsis Campaign
sTREM-1: soluble triggering receptor expressed on myeloid cells-1
TLR: Toll-like receptor
TNF-alpha: tumor necrosis factor-alpha.
