# Supplementary material for: When to initiate immunomodulatory therapy in sepsis
Source: Front Immunol. 2026 Jul 29;17:1898450. doi: 10.3389/fimmu.2026.1898450 (PMC13463184; doi:10.3389/fimmu.2026.1898450)
Supplement: Supplementary file 1 [file DataSheet1.docx]

**Supplementary Tables**

Supplementary Table 1. Clinical value, limitations, and feasibility of major immune-phenotyping tools

| **Tool** | **Immune dimension** | **Signals used in studies** | **Key limitations** | **Practical timing** |
| --- | --- | --- | --- | --- |
| IL-6 / CRP / PCT | Inflammatory burden and infection-related response | No universal cutoff; serial trends are generally more informative than isolated values [3,8,9,31] | Limited specificity; strongly influenced by infection site, pathogen, comorbidity, and sampling time | High feasibility; assess at baseline, 24 h, 48-72 h, and when clinical status changes |
| Ferritin | Hyperinflammation and macrophage activation-like phenotype | PROVIDE and ImmunoSep used >4420 ng/mL as research enrichment criterion [10,11,30] | Not a universal bedside cutoff; affected by liver disease, malignancy, transfusion, hemophagocytic syndromes, and inflammatory comorbidity | Moderate to high feasibility; baseline and repeat testing at 24-72 h when hyperinflammation is suspected |
| mHLA-DR | Monocyte antigen presentation and sepsis-associated immunosuppression | <5000 receptors/monocyte or <8000 mAb/cell used in selected studies [10-14] | Requires standardized flow cytometry; thresholds vary by platform and study | Moderate feasibility in experienced centers; baseline and 48-72 h reassessment are most informative |
| Peripheral lymphocyte count | Adaptive immune dysfunction | Persistent lymphopenia is more informative than a single low value [5,31-33] | Low specificity; affected by stress, corticosteroids, chemotherapy, and underlying disease | High feasibility; daily reassessment in routine ICU practice |
| PD-1 / PD-L1 markers | Checkpoint activation and adaptive exhaustion | No unified clinical threshold; studies focus on dynamic changes or selected enriched populations [15,16,20] | Cell-surface and soluble markers are not interchangeable; assays are not standardized | Low to moderate feasibility; mainly research or experienced centers |
| Functional assays such as LPS-stimulated TNF-alpha release | Innate immune responsiveness and endotoxin tolerance-like hyporesponsiveness | Reduced ex vivo TNF-alpha response indicates impaired innate responsiveness; some studies use TNF-alpha <200 pg/mL to define immunoparalysis [17-24,31] | Sensitive to pre-analytical handling, stimulant dose, incubation time, and assay platform; turnaround time may limit acute decisions | Low to moderate feasibility; research or specialized near-patient platforms |
| Transcriptomic and endotype analysis | High-dimensional immune states and hidden biological heterogeneity | No standardized bedside threshold; mainly used for subgroup discovery or trial enrichment [3,4,17,24,31] | Long turnaround time, high cost, and difficult integration into acute ICU decisions | Low feasibility for current bedside care; research-enhancement tool |

Supplementary Table 2. Therapeutic evidence and timing implications of major immunomodulatory strategies

| **Strategy / evidence** | **Population or enrichment** | **Key findings** | **Implications for initiation timing** | **Evidence positioning** |
| --- | --- | --- | --- | --- |
| Corticosteroids: ADRENAL / APROCCHSS / VANISH [2,25-27] | Adults with septic shock | ADRENAL showed faster shock reversal without clear mortality benefit; APROCCHSS reported lower 90-day mortality; VANISH highlighted interaction with shock management | Most defensible when vasopressor-dependent septic shock persists after appropriate initial management | Guideline-supported near-practice strategy in selected adults with septic shock |
| Anakinra / IL-1 blockade [10,11,28-30] | MALS-like hyperinflammatory phenotype; Shakoory reanalysis, PROVIDE, ImmunoSep | Potential signal in macrophage activation-like phenotype; enrichment designs used high ferritin as a research criterion | Consider only in highly selected hyperinflammatory subgroups or trials; not for unselected sepsis | Biomarker-enriched and investigational outside experienced settings |
| GM-CSF [13] | Low mHLA-DR enrichment in Meisel trial | Improved monocyte immune function and suggested clinical signals | Most relevant when sustained monocyte dysfunction is demonstrated | Small RCT with strong mechanistic signal; not routine care |
| IL-7 [32,33] | Septic shock with lymphopenia in IRIS-7 and subsequent intravenous IL-7 study | Improved lymphocyte counts and selected immune markers | Most relevant in sustained lymphopenia with delayed recovery and adequate infection control | Early-phase clinical evidence |
| PD-1 / PD-L1 blockade [15,16] | Sepsis-associated immunosuppression in selected phase I/II studies | Acceptable early safety and pharmacodynamic immune signals, but no definitive patient-centered benefit | Restricted to research or highly specialized centers | Exploratory strategy |
| IVIG [34,35] | Adult sepsis or septic shock; formulation-dependent studies | Systematic reviews are conflicting; apparent benefit is unstable when restricted to low-bias trials | Does not support routine initiation; possible selected-subgroup relevance remains uncertain | Conflicting evidence; not recommended for routine use |
| Thymosin alpha 1 / TESTS [36] | Adult sepsis in phase III trial | No reduction in 28-day mortality | Illustrates the risk of broad non-enriched immunomodulation | Large negative RCT |

Supplementary Table 3. Translational barriers and feasible countermeasures for implementing immune-guided immunomodulatory therapy in sepsis

| **Barrier** | **Why it affects decisions on timing of initiation** | **Currently feasible countermeasure** |
| --- | --- | --- |
| Insufficient biomarker standardization | Thresholds for the same biomarker differ across platforms and centers [12,14] | Adopt unified standard operating procedures when possible; describe thresholds as enrichment tools rather than universal clinical cutoffs |
| High cost and logistics of serial testing | Dynamic trends rather than single values are most informative for timing decisions [12,22-24,31] | Use tiered monitoring at baseline, 24 h, 48-72 h, and when clinical status changes |
| Limited feasibility in resource-limited ICUs | Repeated flow cytometry or functional assays may not be available [3,12,22,23,31] | Use clinical trajectory, SOFA, lactate, routine inflammatory markers, lymphocyte count, and infection-control status as the first layer of assessment |
| Turnaround time of high-dimensional omics | Results often do not match the acute therapeutic decision window [3,4,17,24,31] | Translate transcriptomic and single-cell discoveries into small surrogate panels and point-of-care combinations |
| Special populations | Pediatric patients, transplant recipients, hematological malignancy patients, and chronically immunosuppressed hosts may not follow adult ICU trajectories [37-39] | Build dedicated cohorts and avoid direct extrapolation of adult algorithms |
| Trial design and ethics | Enrichment designs and adaptive trials require real-time phenotyping and multicenter coordination [8-11] | Use platform trials and embedded biospecimen strategies |
| Overreliance on a single threshold | May miss phenotype transitions or mixed states [3,4,10-14,22-24] | Interpret thresholds together with SOFA, lactate, secondary infection, adverse events, and organ-recovery trajectories |
